# Evaluation of patient satisfaction and maximum biting force of three differently constructed bars on two implants retaining mandibular overdenture - one year follow-up (a randomized controlled clinical trial)

**DOI:** 10.1186/s12903-024-05092-x

**Published:** 2024-11-09

**Authors:** Amr Mohamed Ismail Badr, Mona Nabawy, Gehan Fekry Mohammed, Shaimaa Ahmed Radwan

**Affiliations:** 1 Prosthodontics Department, Faculty of Dentistry, Minia University, Minia, Egypt; 2 Prosthodontics Department, Faculty of Dentistry, Misr University for Science & Technology, Cairo, Egypt; 3 Prosthodontics Department, Faculty of Dentistry, Sinai University, Arish, Egypt

**Keywords:** Bar, Implants, Milled, 3D printed, Patient satisfaction, Maximum biting force

## Abstract

**Background:**

Different bar construction techniques will affect the bar passive fitness, which may induce stresses or strain on the implant and/or tightening screw and sequentially may affect the biting force and patient satisfaction.

**Aim of the study:**

This clinical investigation assessed patient satisfaction and maximum biting force (MBF) using three differently constructed (conventional casting, milling, and 3D printing CAD/CAM techniques) cobalt-chromium (Co-Cr) bar-retained implants mandibular overdentures over a one-year period of follow-up.

**Materials and methods:**

Thirty edentulous patients seeking for two implants bar-retained mandibular overdentures were randomly assigned into three groups as the following: Group I: 10 patients received a Co-Cr conventional casting bar, Group II: 10 patients received a Co-Cr CAD/CAM milled bar, and Group III: 10 patients received a Co-Cr CAD/CAM 3D-printed bar. All the bar groups were connected to two implants in the canine area bilaterally. Within the first two weeks following implant placement, patients received the definitive prosthesis. Patient satisfaction was evaluated by using the (OHIP-EDENT-19) questionnaire form after 6, and 12 months. Additionally, the maximum biting force was tested at after delivery, 3, 6, and 12 months for each group. The results were collected, tabulated, and statistically analyzed. Trial registration: This study was recorded on ClinicalTrials.gov retrospectively registered (ID: NCT06401187) on 30/04/2024.

**Results:**

After one year follow up, regrading patient satisfaction the three groups showed no statistically significant difference. Although, the functional limitation domain was in favor of the milled bar. Regarding the maximum biting force, no statistically significant difference was found among three groups. However, at 12 mouths follow-up the milled bar showed statistically value.

**Conclusion:**

Within the limitations of this study, the conventional, milled and 3D printed bar overdentures groups can be used as a satisfactory treatment modality for edentulous mandible in terms of patient satisfaction and maximum biting force.

## Background

Patients with severely resorbed mandibular ridge frequently experience difficulties wearing conventional dentures. The construction of a conventional complete denture which affords patients good esthetics, masticatory function, and patient comfort with improved stability and retention during function is the most challenging task in dental practice [1].

Implants-retained overdentures have advantages including patient satisfaction, speech, aesthetic improvement, and enhanced stability better than conventional dentures. Implant-retained overdentures may be used for either a variety of splinted bar attachment systems or stud attachments [2].

Bar attachments can enhance prosthesis stability through resisting the lateral forces and limiting the anteroposterior movement which results in improved force distribution and overall overdenture stability [3–5].

Dental alloys have been developed from various materials over the last few years. Compared with other alloys, Co-Cr alloys are inexpensive, and have a high modulus of elasticity, high strength, and high corrosion resistance. Traditionally, conventional cast restorations have many drawbacks, such as shrinkage, expansion, or unintended inclusions during casting, disassembly, intraoral luting, and frequent soldering or laser adjustments to ensure a stress-free fit [6–10].

In contemporary dentistry, Co-Cr dental restorations can be produced using computer-aided design (CAD) and computer-aided manufacturing (CAM) through one of two CAD/CAM processing methods: subtractive manufacturing (SM) or additive manufacturing (AM) [11].

CAD/CAM milling involves subtractive processes to create metallic restorations. By utilizing Co-Cr alloy blanks produced under standardized industrial conditions, the formation of casting-induced flaws and porosities can be minimized. Compared with conventionally produced bars, CAD/CAM milled bars provide high stability levels, compatibility with many implant systems, flexibility in the selection of both therapy and designs a stress-free fit and high precision [10, 11].

Another method for metallic restorations is AM produced by CAD/CAM technology. The desired final restoration is created by applying many layers of material to each other [12]. AM reduces material waste, consumes less energy, has less human intervention and minimizes the number of steps needed to obtain the finished product [13].

The Oral Health Impact Profile (OHIP) is a type of Oral Health-Related Quality of Life (OHRQOL) that is sensitive for detecting patient satisfaction with prosthetic rehabilitation and its subsequent impact on patients’ daily practice [14]. OHIP-EDENT is a modified form (OHIP) for edentulous subjects. The OHIP-EDENT represents 19 questions that has seven domains: functional limitations, physical pain, psychological discomfort, physical disability, psychological disability, social disability, and handicap. It assesses the levels of dysfunction, discomfort, and disability in a person’s quality of life [15].

The Maximum Bite Force (MBF) is the maximum force released by the lower teeth against the upper teeth which is responsible for the upward movement of the mandible [16, 17]. Biting force measurement is a broad method used to assess dental prosthesis function and efficacy, that involves the use of different devices and methods [18].

Hence, the goal of this randomized clinical study was to prospectively monitor patient satisfaction (OHIP-EDENT-19 questionnaire form) and maximum biting force outcomes in three differently constructed bars over a one-year follow-up period. The first bar was casted by a conventional method, the second was a milled bar (subtractive processes), and the third was a 3D printed bar (additive processes); both the second and third were fabricated by CAD/CAM Digital Technology.

The null hypothesis assumes there is no significant differences among the three groups regarding patient satisfaction and maximum biting force.

## Materials and methods

### Study design

This study was set to be a single-blind, randomized clinical trial according to the Consolidated Standards of Reporting Trials (CONSORT) rules for clinical trials [19]. Microsoft excel was used for patient allocation into three equal parallel groups at a 1:1:1 allocation ratio.

Thirty patients received two implants retained with bar mandibular overdentures; Group I: conventional casting bar (control group), Group II: milled CAD/CAM bar, and Group III: 3D printed CAD/CAM bar.

### Ethical approval and protocol registration

The study protocol was registered at www.clinicaltrials.gov (ID NCT06401187). All procedures involving human participants were done following the ethical standards of the Research Ethics Committee of the Faculty of Dentistry and was reported with a specific serial number of 644 (Ref. no. 25 / 10 / 2022). All the patients were informed about all treatment details and signed a consent form.

### Sample size calculation

The sample size was calculated to detect patient satisfaction (as a primary outcome) among the studied groups, based on a previous study [20]. The sample size was calculated using G- Power version 3.1.9.2 [21] adopting a power of 80% (β = 0.20) to detect a standardized effect size of 0.684 and at a level of significance of 5% (α error accepted = 0.05), the minimum required sample size was found to be 8 patients per group (number of groups = 3) (total sample size = 24 patients) [22, 23]. After considering a dropout rate of 10%, the sample size was increased to 10 patients per group (total sample size = 30 patients) [24].

### Randomization and blinding

Thirty participants were randomly assigned to either control, milled or 3D printed groups using block randomization in Microsoft Office Excel 2010 (Microsoft Corporation, Redmond, WA, USA), and the assignments were documented on a printed table. Eligible patients were sequentially assigned with specific numbers, and to maintain allocation concealment, access to the table was restricted to a designated co-investigator who disclosed each patient’s assigned group upon request [25].

### Patient selection

**Fig. 1:**
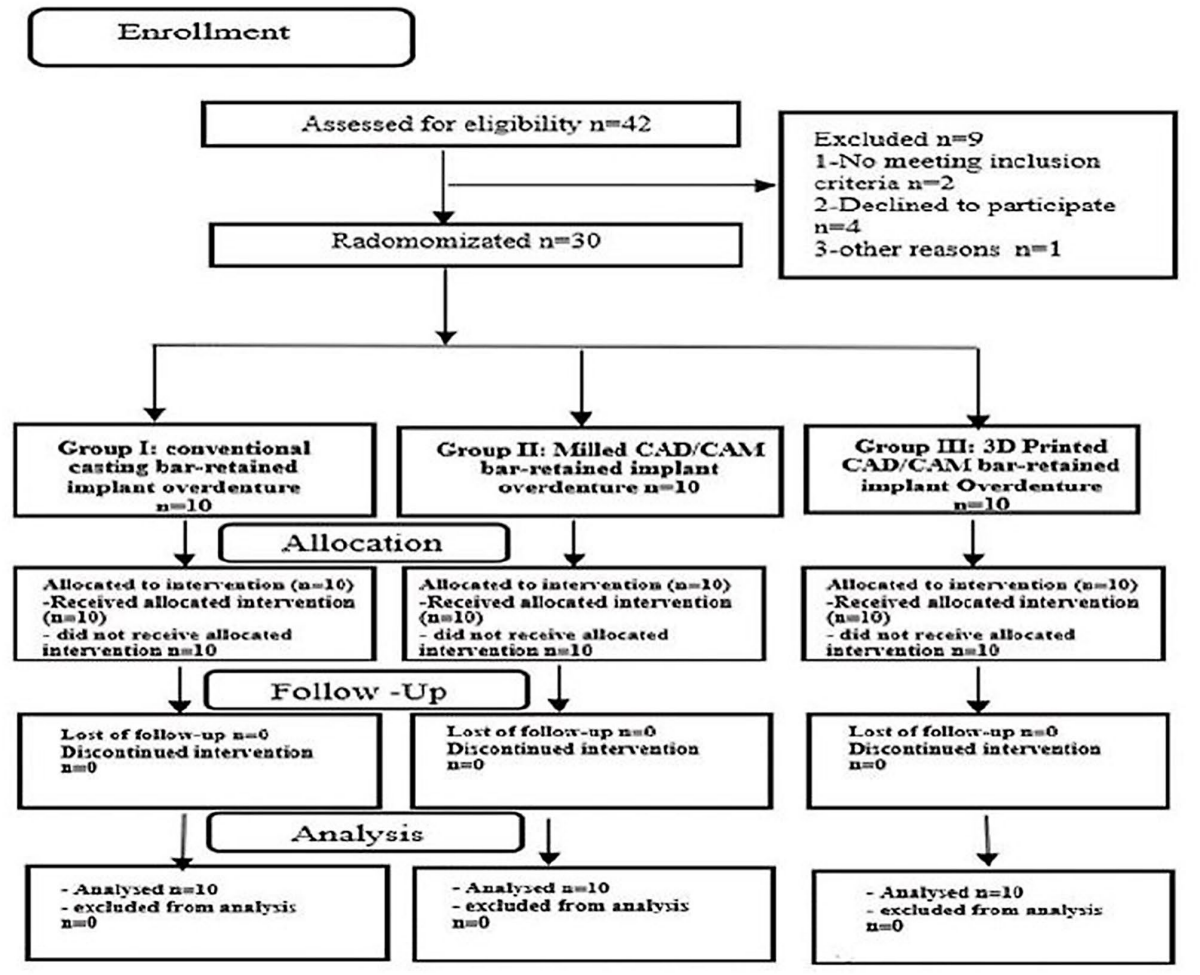
Study flow diagram following the CONSORT guidelines

Patients with completely edentulous maxillary and mandibular arch who seek bar-retained two implants mandibular overdenture were selected from the outpatient clinic of the Prosthodontic Department, at the Faculty of Dentistry, Minia University. All patients were selected according to the inclusion criteria in this study (Table 1) Fig. 1.

**Table 1:** Inclusion and exclusion criteria

Inclusion Criteria	Exclusion criteria
1. Provision of informed consent.	1. It is unlikely to be able to comply with the study procedures.
2. Patients with sufficient residual alveolar bone quantity (height and width) and quality (normal trabecular pattern) anterior to the mental foramen to receive self-taping root form titanium implants.	2. Patients with systemic diseases such as cardiovascular diseases, any disease of immunity, febrile conditions such as epilepsy, metabolic disorders, osteoporosis, hyperparathyroidism, and impaired psychological conditions that might affect the oral tissues or the bone metabolic rate.
3. Maxillary and Mandibular residual alveolar ridges covered with healthy mucosa without any remaining roots or local inflammation.	3. Patients with local and general contraindications for surgical procedures.
4. Patients with sufficient inter-arch space (at least 15 mm) to have overdenture and bar attachment.	4. Patients with TMJ or neuromuscular disorder.
5. U-shaped lower ridge to avoid the lingual placement of the bar that occurs with a V-shaped ridge.	5. Patients with Para functional habits such as bruxism and clenching.
	6. Patients with a history of bisphosphonate intake.

### Clinical procedure

All patients received a complete denture construction, following the conventional procedures. Patients received instructions on proper denture and oral hygiene measures after maxillary and mandibular complete dentures. Any adjustments to eliminate occlusal discrepancies and ensure patient satisfaction were also carried out.

The existing mandibular denture was duplicated twice with a clear self-cure acrylic resin to serve as a radiographic stent, and the other to serve as a surgical template for implant placement using agar-agar material in a duplicating flask.

Gutta-percha markers were added (Meta Biomed, Chungcheong Buk, South Korea) to the fitting surface of the mandibular radiographic stent. Cone Beam Computed Tomography (CBCT) (Scanora 3d, soredex. Nalikelantie 160, P.O.Box 148, FI-04301 Tuusula. Finland) scan was performed for every patient while wearing the radiographic stent.

### Surgical protocol

Implant placement surgery was performed. First, a bleeding point was created through the surgical stent to ensure the appropriate positioning of the osteotomy sites. Osteotomy preparation was performed using a horizontal flap at the crest of the ridge (open hand technique).

**Fig. 2:**
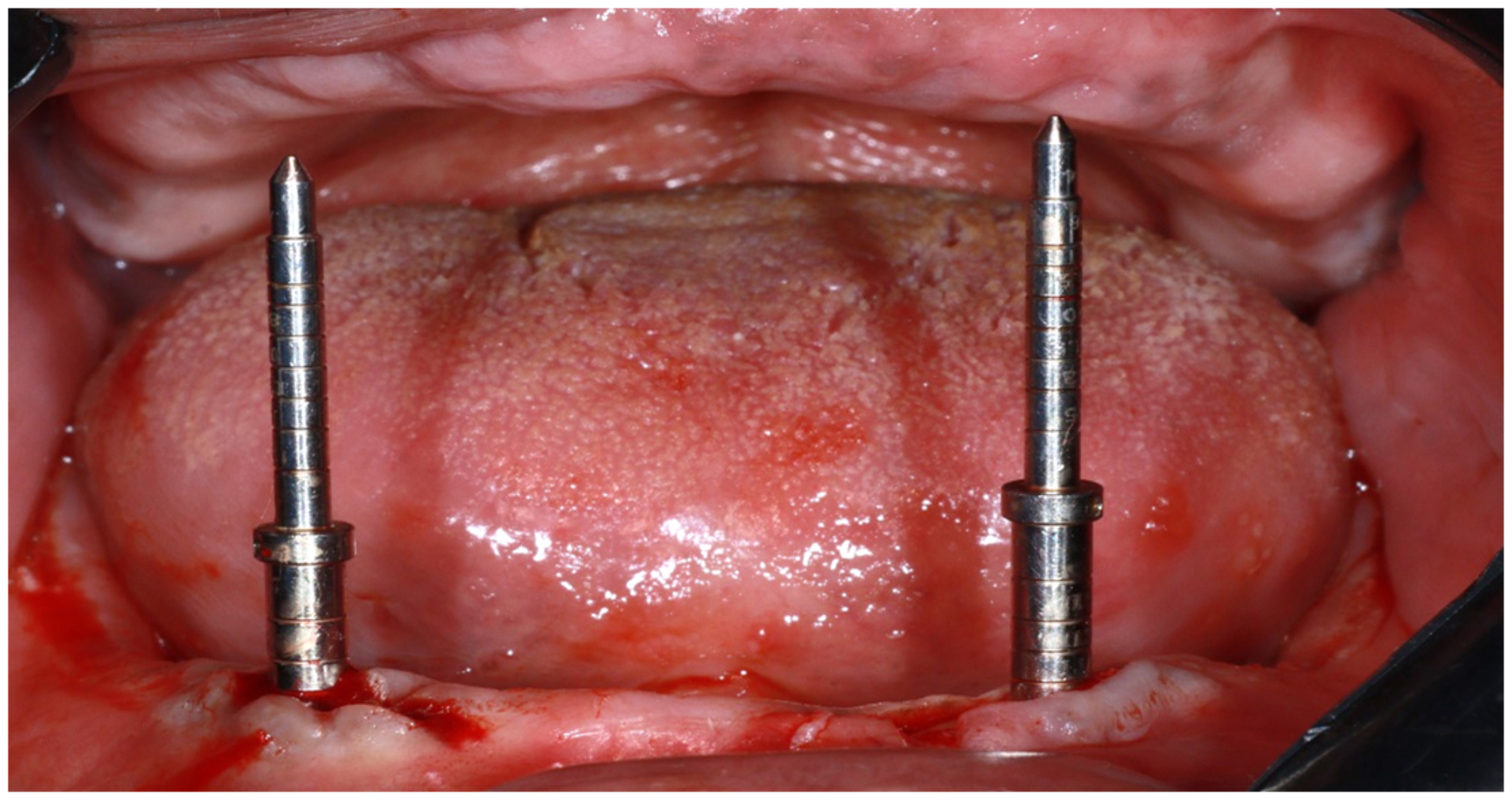
Paralleling pins are used to verify and maintain the correct alignment and orientation of the drill during preparation at osteotomy site

Sequential drilling was performed through copious saline irrigation according to the drilling protocol [26]. The parallelism was checked using parallel pins. Figure 2 Dental implants (Vitronix, TURATI,38 20121 MILANO-ITALY) with diameter of 3.7 mm and a length of 13 mm were placed in the osteotomy site. The implants were seated to be flushed with crestal bone using a torque wrench, implants were tightened with a torque of 35–40 N/cm.

Straight multi-unit abutments were installed and screwed manually by a torque wrench with a torque of 10–15 N/cm. The flap was closed using an interrupted suturing technique. After surgery, the patients were advised to apply a cold pack for twenty minutes every 1 h for the rest of the operation day. Proper antibiotic program, analgesic and anti-inflammatory medication were prescribed.

To further optimize comfort during the healing phase and before the definitive denture is delivered, mandibular denture opposite the surgical site should be relieved bilaterally, and tissue conditioning material (Dental GC Tissue Conditioner Soft acrylic tissue conditioning and relining -TOKYO- JAPAN) should be applied that should be changed every 72 h, to act as a cushion beneath the denture.

### Prosthetic protocol

#### Bar fabrication

For bar fabrication, an open tray impression technique was performed one week after the surgery [27]. To ensure implant immobility a verification jig was made [28]. Cast was scanned with a desktop scanner (DOF, SHINING 3D, South Korea) to obtain the bar design.

**Fig. 3:**
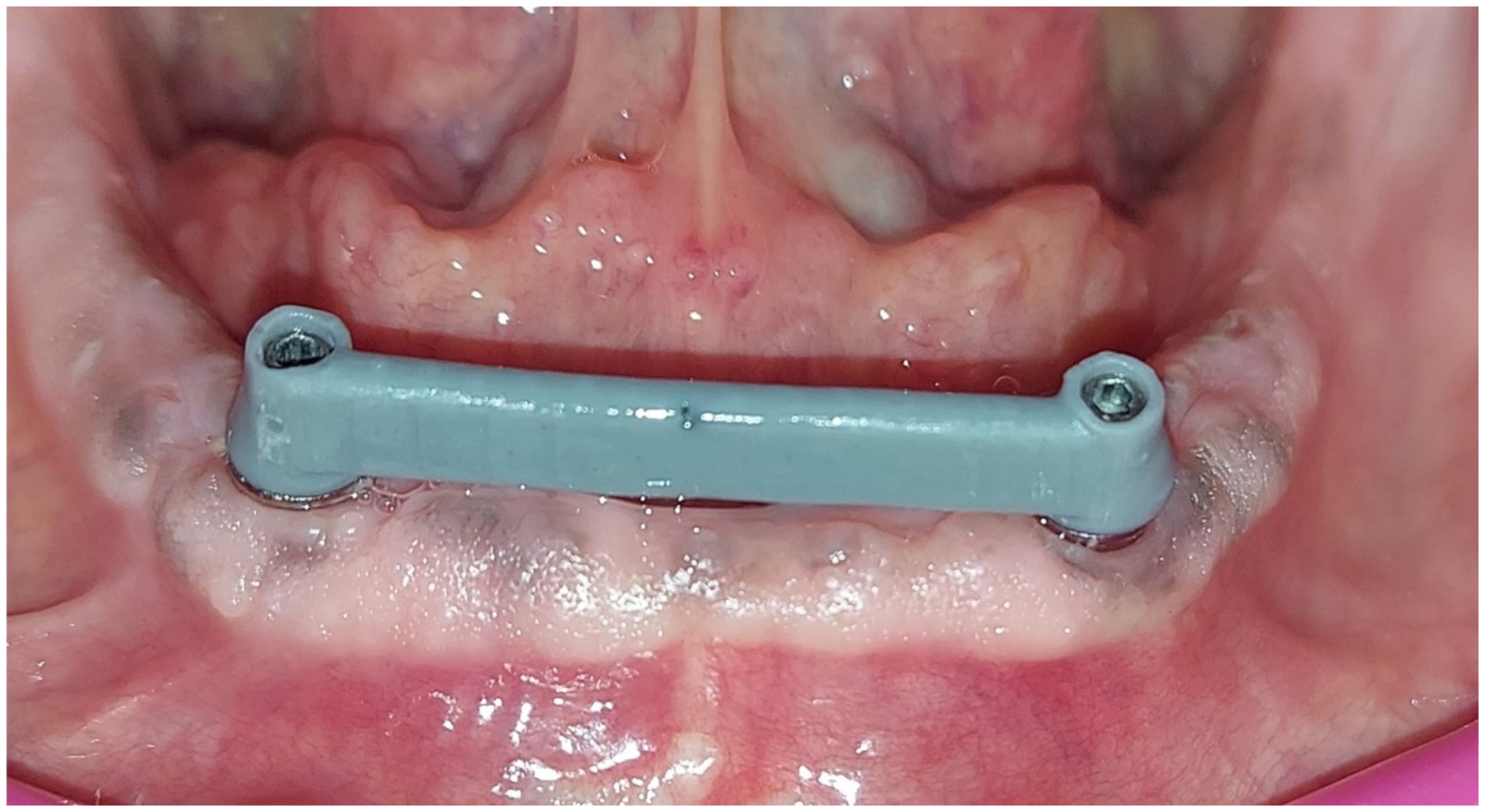
PMMA bar try-in in patient mouth

Using software (Exocad GMBH, Germany), the selected bar design was OT castable Bar (Rhein83, Italy) design, the bar had a rounded top cross section and flat bottom with dimension of 2 mm width and 4 mm height [29]. A polymethyl methacrylate (PMMA) bar try-in was constructed and checked inside the patient’s mouth for passive fitness, and extension for each bar type. Finally, the bar is ready for casting, milling, or 3D printing according to patient enrollment Figure 3.

### Group I: control group (conventional bar construction)

**Fig. 4:**
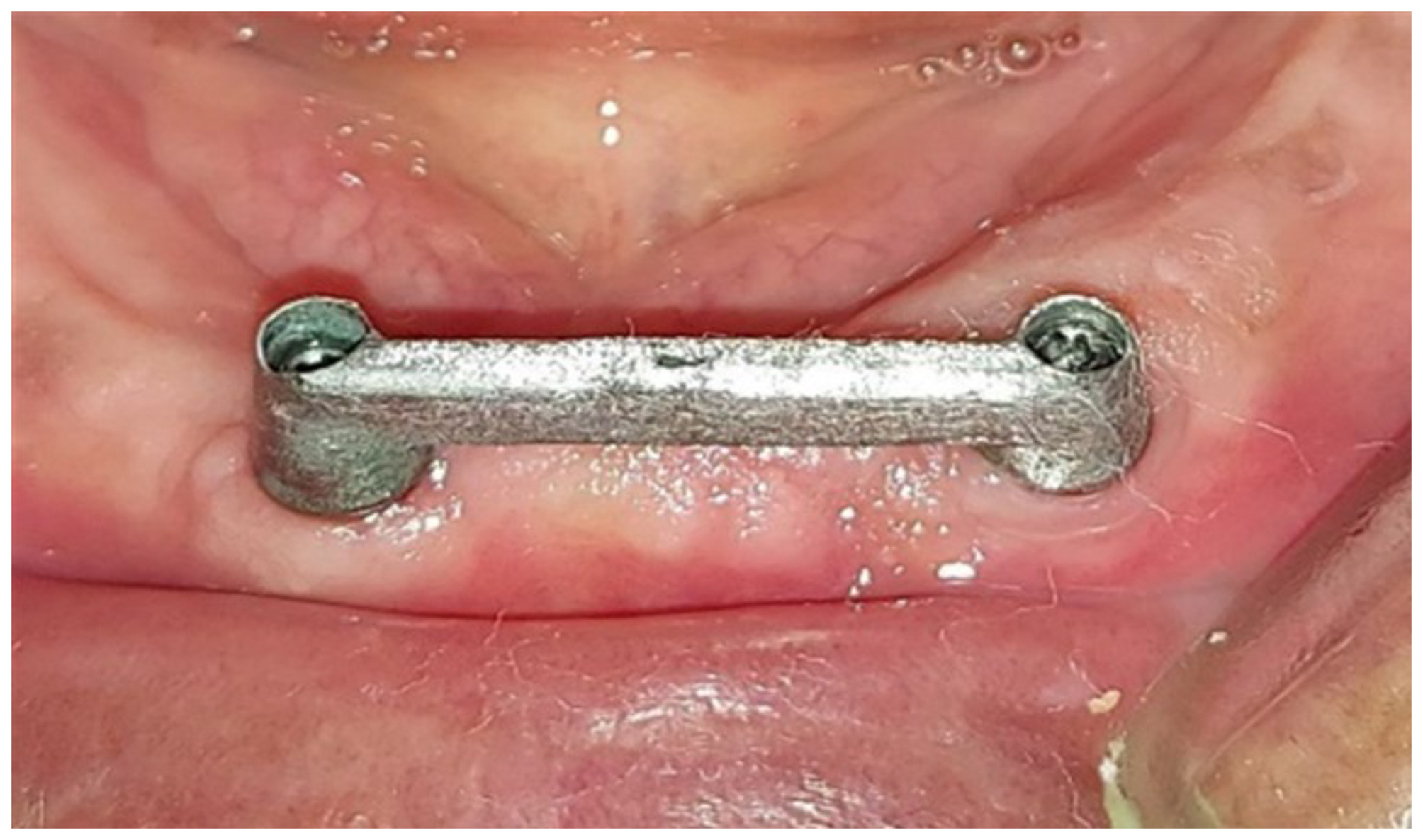
Conventional cast Co-Cr bar-retained implant

After the PMMA try-in bar was checked a wax bar was 3D printed. The wax bar was transferred to a metal Co-Cr alloy (BEGO Medical Gmbh, Bremen, Germany) bar using a conventional casting technique. Finished and polished according to manufacturer’s instructions Figure 4.

### Group II, and Group III (CAD/CAM bars construction)

After the design was checked, the Standard tessellation language (STL) file was exported to a milling machine (ED5X- EMAR, Egypt), or 3D printing machine (VULCANTECH, Germany) CAD/CAM techniques.

Group II utilized a milled Co-Cr block (Mogucera C disc, Germany) for the bar, which was crafted using a computer milling machine (CAM) (Ceramal, Amann Girrbach, Austria).

**Fig. 5:**
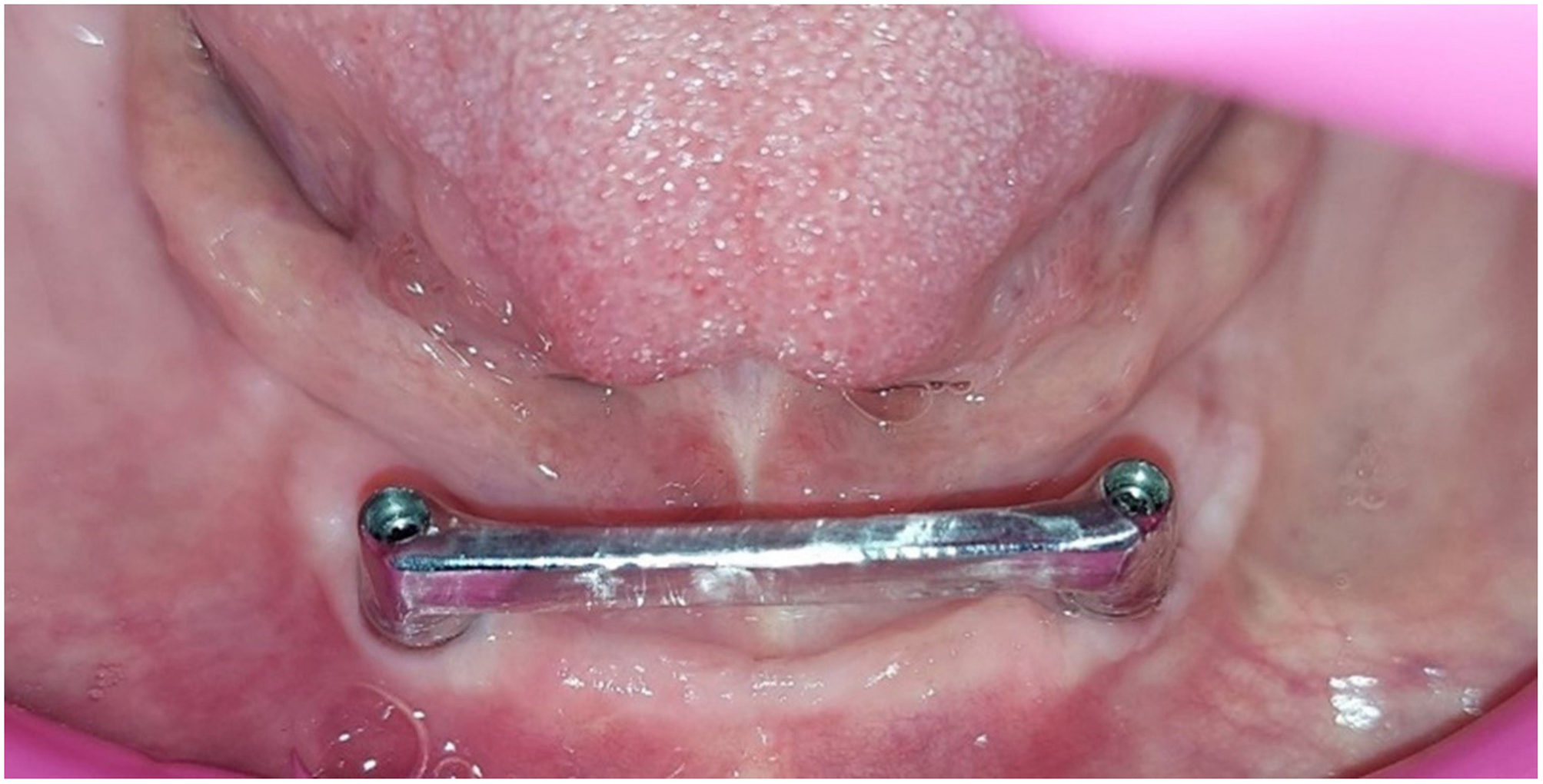
CAD/CAM milled Co-Cr bar-retained implant

The milling process involved gradual removal of excess material by cutting tools to shape the bar as per the predetermined CAD design Figure 5.

**Fig. 6:**
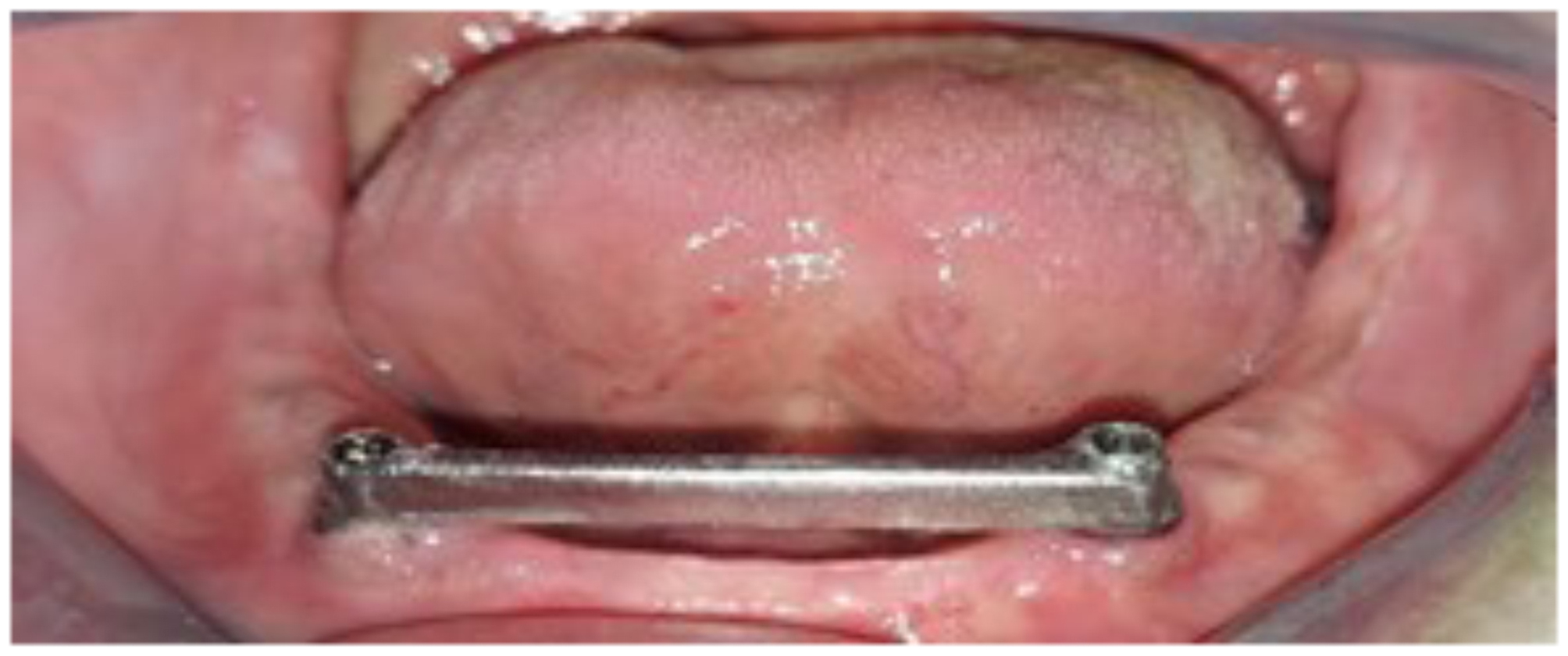
CAD/CAM 3D Co-Cr bar-retained implant

Group III employed selective laser melting (SLM), a 3D printing technique. The process began by placing raw powdered Co-Cr material into a tray, and then using a laser beam (HBD 100D, Guangdong Hanbang 3D Tech Co. Ltd., China) to manufacture the bars via 3D printing (SLM). The laser beam was directed over the tray to heat the powdered material layer by layer, effectively binding the particles into the desired shape Figure 6.

### Modification of the mandibular overdentures

Tightening the bar to the implants and starting modification of mandibular overdentures, by rebasing technique.

### Loading protocol

After two weeks of surgery, the Co-Cr bar was placed according to the immediate loading protocol (ILP).

The construction of the overdenture was completed in the usual manner. For the pick-up procedures, a plastic clip (OT bar Rhein83, Bologna, Italy) was attached to the bar, and sticky wax was used to block out the undercut beneath the bar on the day of delivery. Pick-up of the clip was made directly in the patient’s mouth. A space was created for the clip to pick up the space on the fitting surface of the prostheses opposite the clip, and escape holes on the lingual side of the denture were opened, then the space was filled with Auto polymerizing acrylic resin.

Finally, the mandibular overdenture was rechecked for occlusal imperfections and was delivered to the patient. Patients were scheduled for follow-up appointments to check for any complaints.

### Patient satisfaction measurement

**Fig. 7:**
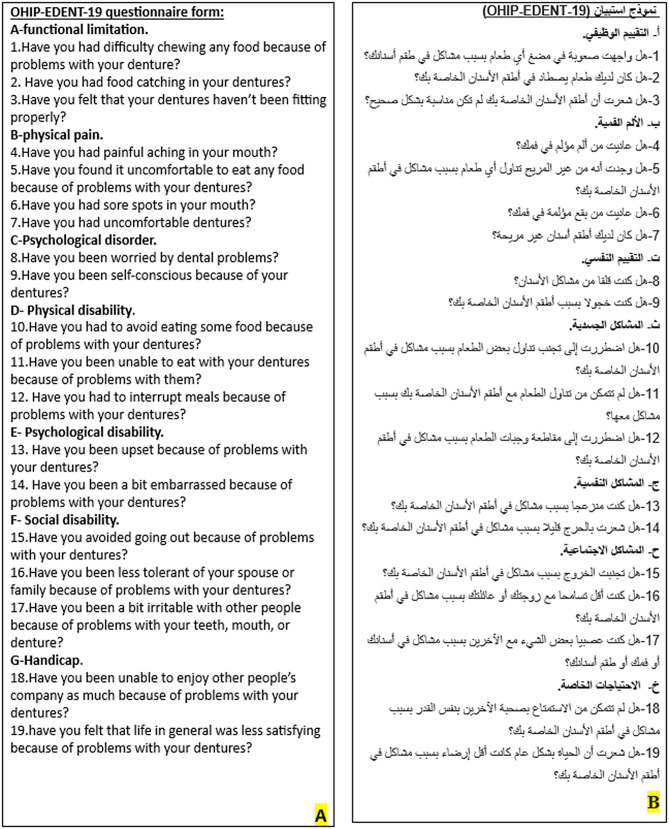
**A**- OHIP-EDENT-19 questionnaire form, **B**- Arabic translation form

Patient satisfaction with the definitive prosthesis was assessed using the OHIP-EDENT-19 questionnaire and evaluated at 6 months, and 12 months after delivery. The mentioned questionnaire was translated from English to Arabic language to address all sociodemographic categories easily Figure 7. All participants listened to the Arabic translation from a co-investigator, and questionnaire’ scores of questions responses were never (= 1), hardly ever (= 2), occasionally (= 3), fairly often (= 4), and very often (= 5). Lower scores indicate higher satisfaction, higher scores indicate lower satisfaction [30, 31].

### Maximum Biting Force (MBF) measurement

Maximum Biting Force (MBF) was assessed using the occlusal force meter device (GM10, Nagano Keiki, Tokyo, Japan) and evaluated at after delivery, 3, 6, and 12 months.

**Fig. 8:**
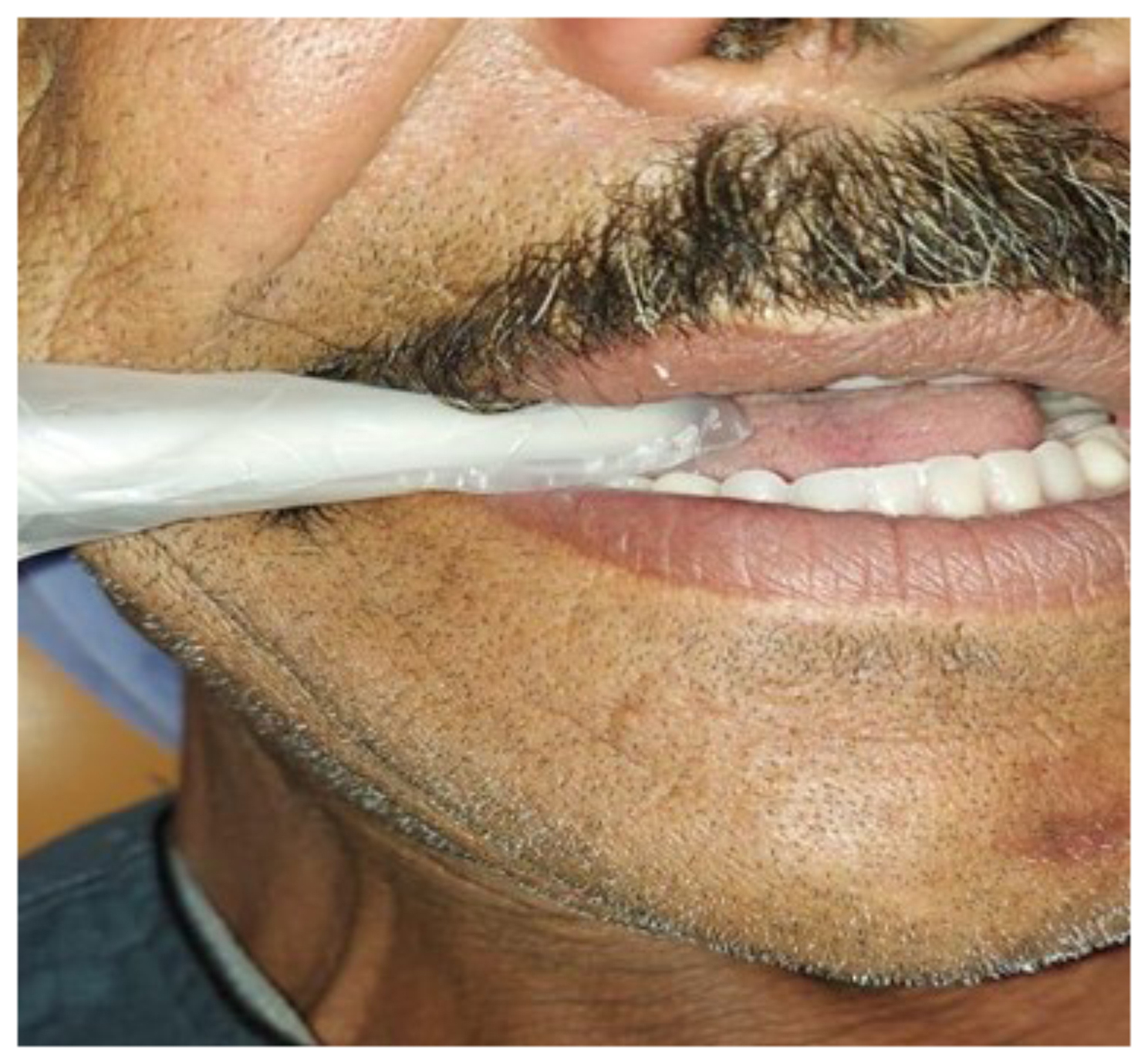
Evaluation of MBFs by the occlusal force-meter device in the patient’s mouth

During the measurements, the arm of the occlusal force meter sensor device, was enclosed in a plastic tube, positioned in patient’s mouth at the first molar tooth on both sides. Patients were instructed to bite down slowly on the device, recording the maximum biting force on each side at 30-second intervals. This process was then repeated five times measurements on each side, and scores were collected and average to obtain a single score for each side. Patients sustained biting force levels throughout the testing, with a buzzer alerting me to record the readings of the maximum biting force levels. The maximum biting force (MBF) readings were digitally displayed in Newton units on the digital device screen. Measures were recorded at a state free of pain or discomfort [32] Figure 8.

Data was collected, tabulated, and statistically analyzed. The data are presented as the means, standard deviations, ranges, or frequencies (number of patients) and percentages.

### Data analysis

Statistical analysis was performed; the OHIP-EDENT-19 questionnaire form scores which are non-normally distributed (non-parametric) data and Maximum biting force data showed normal distribution (parametric). The parametric data was analyzed using repeated measures ANOVA to compare the three groups and evaluate changes over time within each group. Pair-wise comparisons were conducted using Bonferroni’s post-hoc test when ANOVA results were significant. Non-parametric data were assessed using the Kruskal-Wallis test across the three groups, with Dunn’s test employed for pair-wise comparisons if the Kruskal-Wallis test yielded significance. Qualitative data were reported as percentages and frequencies. The Fisher’s Exact test compares differences among the three groups. Statistical significance was set at *P* ≤ 0.05.

## Results

The study was completed without a drop out (survival rate 100%).

### Patient satisfaction questionnaire (OHIP-EDENT-19 questionnaire form)

#### After six months

There was a statistically significant difference in the functional limitation domain between the milled bar group and the other bar groups (P value = 0.003). Among the groups, pair-wise comparison revealed that there was no statistically significant difference between conventional and 3D printed bar groups; as both groups had statistically significantly higher scores than milled bar group.

There was a statistically significant difference between the 3D bar group and the other bar groups regarding the physical disability domain (P-value = 0.040). Among the groups, pair-wise comparisons revealed that there was no statistically significant difference between the conventional and milled bar groups, as they both lower scores than did the 3D bar group.

There was a statistically significant difference between the three groups regarding the total score (P-value = 0.016). Among the groups, pair-wise comparison revealed that the conventional bar group showed the statistically significantly highest score. 3D printed bar group showed a statistically significantly lower score. The milled bar group showed statistically significantly lowest score.

**Fig. 9:**
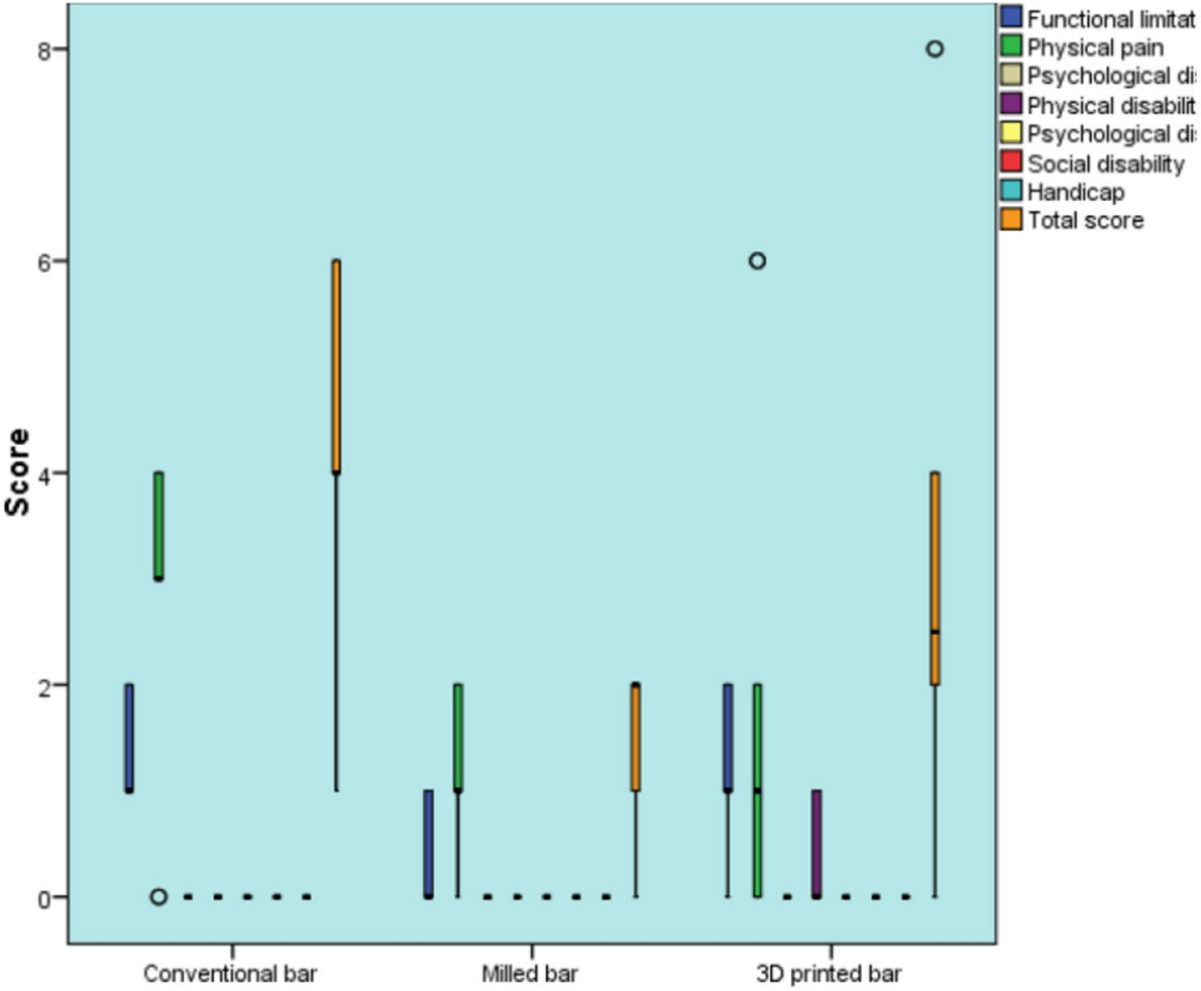
Box plot representing the median and range values for the OHIP-EDENT-19 questionnaire scores in the three groups after six months (circles represent outliers)

There was no statistically significant difference among the three groups regarding all other dimensions (Table 2) Fig. 9.

**Table 2:** Descriptive statistics and results of the Kruskal-Wallis test for comparison between OHIP-EDENT-19 questionnaire scores in the three groups after six months in different domains

Domains	Conventional bar (*n* = 10)		Milled bar (*n* = 10)		3D printed bar (*n* = 10)			Effect size (Eta squared)
	Median (Range)	Mean (SD)	Median (Range)	Mean (SD)	Median (Range)	Mean (SD)	*P*-value	
Functional limitation	1 (1,2)^A^	1.3 (0.5)	0(0, 1)^B^	0.3 (0.5)	1 (0, 2) ^A^	1.1 (0.7)	0.003*	0.381
Physical pain	3 (0, 4)	2.7 (1.5)	1 (0, 2)	l.l(0.7)	1 (0. 6)	1.8 (2.4)	0.067	0.144
Psychological disorder	0(0, 0)	0(0)	0 (0, 0)	0(0)	0 (0, 0)	0(0)	1	0
Physical disability	0 (0,0)^®^	0(0)	0(0, 0)^B^	0(0)	0 (0, 1) ^A^	0.3 (0.5)	0.040*	0.222
Psychological disability	0(0, 0)	0(0)	0 (0, 0)	0(0)	0 (0, 0)	0(0)	1	0
Social disability	0(0, 0)	0(0)	0 (0, 0)	0(0)	0 (0. 0)	0(0)	1	0
Handicap	0(0, 0)	0(0)	0 (0, 0)	0(0)	0 (0. 0)	0(0)	1	0
Total score	4(1, 6)^A^	4(1.8)	2(0, 2) ^c^	1.4 (0.8)	2.5 (0, 8) ^B^	3.2 (2.8)	0.016*	0.247

*Significant at *P* ≤ 0.05, A, and B superscripts in the same row indicate statistically significant differences between groups

#### After 12 months

**Fig. 10:**
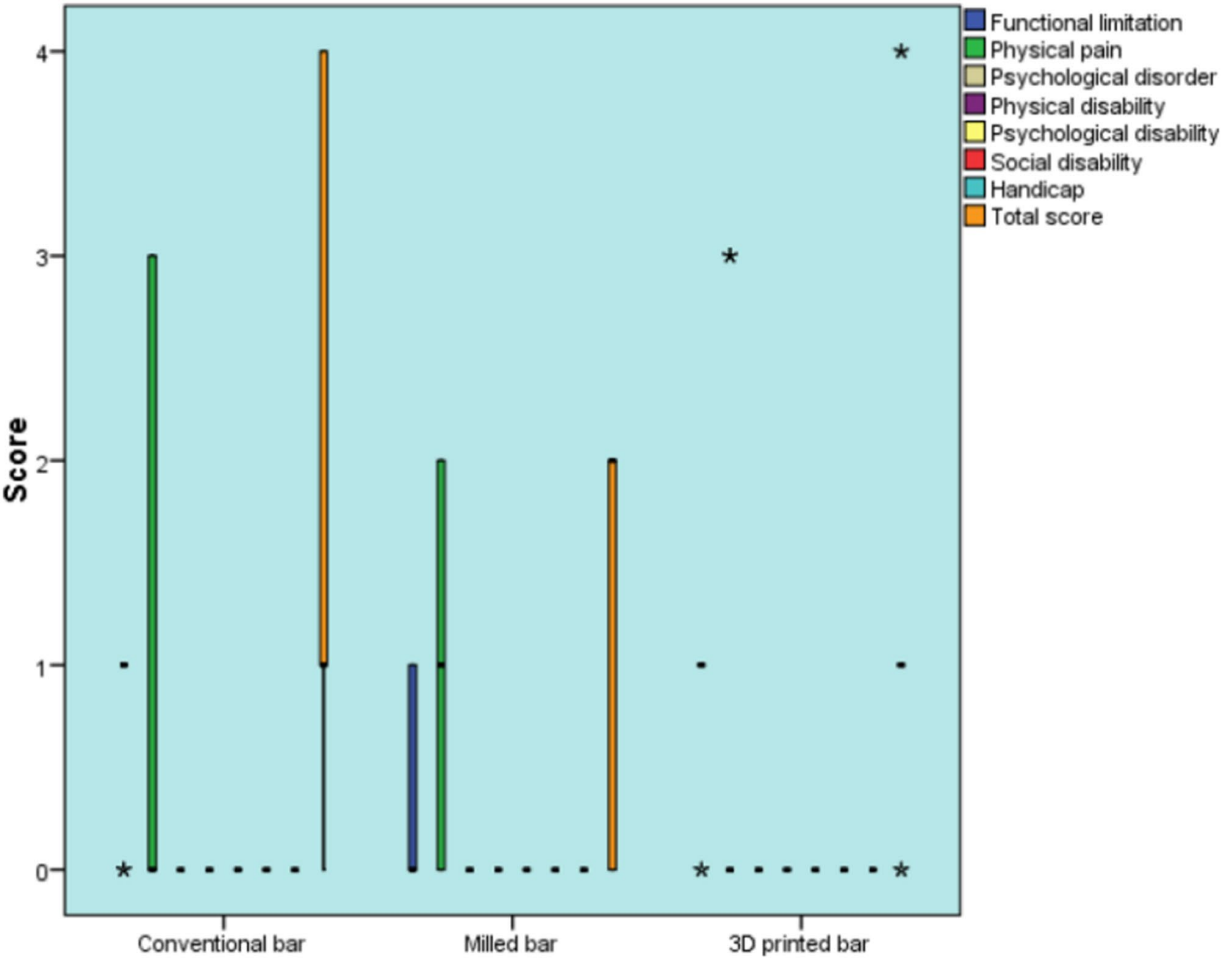
Box plot representing median and range values for OHIP-EDENT-19 questionnaire scores in the three groups after 12 months (stars represent outliers)

- There was a statistically significant difference between the milled bar group and the other bar groups regarding the functional limitation domain (P- value = 0.031). Among the groups, pair-wise comparison revealed that there was no statistically significant difference between the conventional and 3D printed bar groups, as they both showed statistically significantly higher scores than did the milled bar group.
- There was no statistically significant difference among the three groups regarding all other dimensions as well as the total score (Table 3) Fig. 10.

**Table 3:** Descriptive statistics and results of Kruskal-Wallis test for comparison between OHIP-EDENT-19 questionnaire scores in the three groups after 12 months in different domains

Domains	Conventional bar (*n* = 10)		Milled bar (*n* = 10)		3D printed bar (*n* = 10)		*P*-value	Effect size (Eta squared)
	Median (Range)	Mean (SD)	Median (Range)	Mean (SD)	Median (Range)	Mean (SD)		
Functional limitation	1 (0. 1)^A^	0.8 (0.4)	Ho. D^B^	0.3 (0.5)	1(0, 1)^A^	0.8 (0.4)	0.031*	0.239
Physical pain	o (0, 3)	0.9 (1.4)	1 (0,2)	0.9 (0.9)	0 (0, 3)	0.6 (1.3)	0.515	0.015
Psychological disorder	0 (0, 0)	0(0)	0(0,0)	0(0)	0(0.0)	0(0)	1	0
Physical disability	0 (0, 0)	0(0)	0 (0, 0)	0(0)	0 (0, 0)	0(0)	1	0
Psychological disability	0 (0. 0)	0(0)	0(0,0)	0(0)	0(0,0)	0(0)	1	0
Social disability	0 (0. 0)	0(0)	0(0,0)	0(0)	0(0,0)	0(0)	1	0
Handicap	0 (0, 0)	0(0)	0(0,0)	0(0)	0(0.0)	0(0)	1	0
Total score	1 (0. 4)	1.7 (1.6)	2(0,2)	1-2(1)	1 (0,4)	1.4 (1.4)	0.930	0.024

*Significant at *P* ≤ 0.05, A, and B superscripts in the same row indicate statistically significant differences between groups

### Comparison of the maximum biting force among the three groups

- At the onset of the statistical analysis, the maximum biting force was measured on both sides (right and left first molars). No statistically significant difference was observed between the two sides individually. Therefore, we calculated the mean value by summing the results from both sides for each patient.
- After overdenture delivery (*P*-value < 0.001) as well as after three months (*P*-value < 0.001), there was a statistically significant difference between maximum biting force among the three groups. Pair-wise comparisons between groups revealed that the milled group showed the statistically significantly highest mean maximum biting force value, followed by the 3D printed bar group. The conventional bar group had the least significant mean maximum biting force.

After six months (*P*-value = 0.374) as well as 12 months (*P*-value = 0.374), there was no statistically significant difference between maximum biting force values in the three groups.

### Changes in maximum biting force within each group (effect of time)

**Fig. 11:**
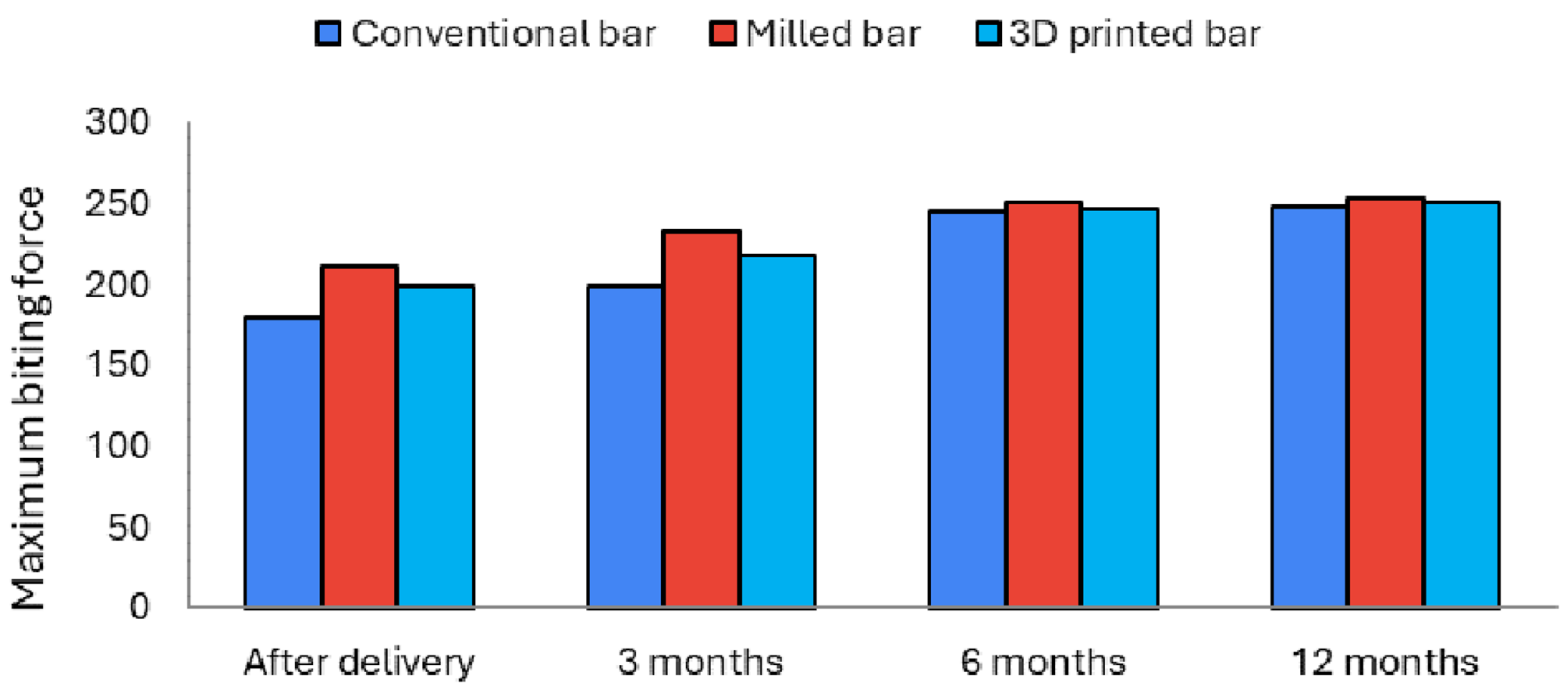
Bar chart representing the mean and standard deviation values for the maximum biting force values in the three groups

- In the conventional and the 3D printed bar group: there was a statistically significant change in the maximum biting force values by time (*P*-value < 0.001). Pair-wise comparisons between periods revealed that there was a statistically significant increase in maximum biting force at overdenture delivery as well as at three months. However, there was no statistically significant difference in the maximum biting force between six months and 12 months.
- In the milled bar group: there was a statistically significant change in the maximum biting force over time (*P*-value < 0.001). Pair-wise comparisons between periods revealed that there was a statistically significant increase in the maximum biting force at overdenture delivery. However, there was no statistically significant difference in maximum biting force between three months, six months, and 12 months (Table 4) Fig. 11.

**Table 4:** Descriptive statistics and results of repeated measures ANOVA test for comparison between maximum biting force values of first molar teeth in the three groups and the changes within each group

Time	Conventional bar (*n* =10)		Milled bar (*n* =10)		3D printed bar(*n*=10)		*P*-value	Effect size (Partial Eta squared)
	Mean	SD	Mean	SD	Mean	SD		
After delivery	1SO^CT^	25	211“	14	199“	11	<0.001*	0.921
3 months	198^13^	9	22*0	12	218“	13	<0.001*	0.592
6 months	245 °	8	250 ^D^	9	246°	7	0.374	0.042
12 months	248 ^D^	11	2530	12	251 ^D^	11	0.344	0.069
P-value	<0.001*		<0.001*			<0.001*		
*Effect size (Partial Eta squared)*	0,935		0.837			0.911		

* Significant at *P* ≤ 0.05, A, B, and C superscripts in the same row indicate statistically significant differences between groups, D, E, and F superscripts in the same column indicate statistically significant change by time

## Discussion

This clinical investigation assessed patient satisfaction and maximum biting force (MBF) using three differently constructed (conventional casting, milling, and 3D printing CAD/CAM techniques) cobalt-chromium (Co-Cr) bar-retained implants mandibular overdentures over a one-year period of follow-up.

In the current study there was no significant difference regarding patient satisfaction and maximum biting force among three bar groups. Hence, the null hypothesis was accepted.

Souza RF et al., used the OHIP-EDENT-19, is a validated questionnaire designed for edentulous patients [33]. Their findings indicate that this questionnaire can be used to effectively track oral health changes over time and estimate the impact of oral treatments [34]. In our study, we translated the OHIP-EDENT-19 into Arabic language to improve patient comprehension of the questionnaire.

In the current study, after one year follow-up there was no statistically significant difference between the three bar groups. Although, the functional domain was in favor of the milled bar group. In a clinical crossover study comparing zirconia bar and cobalt chromium bar retaining mandibular implant overdenture no significant difference between the two groups was reported with accepted outcomes of speech, chewing capacity, restorative procedures, complications and information prior to treatment regarding the cast Co-Cr bar group which is in one line with our study. However, the zirconia bar group reported a higher esthetically accepted prosthesis over the casted bar group [35].

Furthermore, in a crossover clinical trial evaluating patient satisfaction between conventional denture, stud overdenture, telescopic and bar overdenture. The casted bar overdenture group showed higher patient satisfaction during biting hard and soft foods which agreed the result of the current study [29].

In a systemic review comparing different clinical aspects between various attachments system (bar, locator, magnets, Hader clips, ERA, telescopic crowns, and OT equator) attachments, bar attachment showed higher patient satisfaction among the other attachment system [36].

In addition, in a systematic review and meta-analysis evaluating patient satisfaction, survival rate and prosthetic maintenance between four and six implants bar retained mandibular overdentures. A satisfactory performance for each group was reported with non-inferiority of both treatment modalities [37].

Maximum biting forces (MBFs) are critical for masticatory function evaluation [38]. MBF has different values in various locations in the oral cavity, and it is commonly recorded in the first molar region since most of the biting force is directed concentrated in this area [39, 40]. In this study, the maximum biting forces were measured at five different records on each patient’s first molars bilaterally to ensure accurate data and measurements collection. This approach aligns with the findings of Ikebe K et al. findings, who suggested that multiple recordings provide more reliable results than a single measurement [41].

Measurements of biting force across the three different constructed bars indicated no significant difference between the right and left sides. This can be attributed to all patients involved in this study that don’t have a preferable chewing side. Additionally, patients used both sides eventually while eating with their bar-retained two implants mandibular overdenture.

The range for measuring maximum bite force (MBF) was from 0 to 1000 N, with an accuracy of ± 1 N. Sharma et al., reported that complete denture wearers typically exerted masticatory forces ranging from 60 to 80 N, whereas patients with implant-supported mandibular overdentures demonstrated greater forces, ranging from 150 to 170 N [42].

The biomechanics of the jaw elevator muscles, and the lever system of the mandible cause the maximum bite force on the molars compared with the incisors. The number of occlusal contacts significantly influences muscle action and bite force more than the count of individual teeth. This connection implies that adequate occlusal support can strengthen elevator muscles, resulting in greater biting force and enhanced functional performance [43].

In a crossover study, evaluating chewing efficiency and maximum bite force (MBF) between telescopic and bar implant retained overdentures, the results showed an increase in maximum biting force results by time regarding bar overdenture group. However, the telescopic group showed increased MBF values for telescopic attachment overdenture which is in one line with our study [44].

In crossover clinical study evaluating maximum biting force (MBF) between conventional dentures (CD), fixed prostheses (FR), and milled bar overdentures (MO) stating that the highest MBF values were recorded in milled bar overdenture over fixed prosthesis and conventional denture, which agreeing with the result of our study [45].

In addition, a randomized clinical trial between CAD-CAM milled bars with distal extensions and retentive anchors in two implants retained overdentures, evaluating maximum biting force. Although, there was no significant difference between the two groups. Yet, the milled bar group exhibited increase MBF values by time which was comparable with result of our study [46].

However, the study had some limitations, including a small sample size and a short follow-up period of just one year. Further clinical research with longer follow-up periods and larger sample sizes are necessary to evaluate the patient satisfaction and MBF of milled and 3D printing bar overdenture more effectively.

## Conclusions

Within the limitations of this study, the conventional, milled and 3D printed bar overdentures groups can be used as a satisfactory treatment modality for edentulous mandible in terms of patient satisfaction and maximum biting force.

## Data Availability

The data during the current study are available from the corresponding author on reasonable request.

## Abbreviations

CBCT: Cone-Beam Computed Tomography
ILP: Immediate Loading Protocol
3D: Three-Dimensional
CAD–CAM: Computer-Aided Design and Computer-Aided Manufacturing
AM: Additive Manufacturing
SM: Subtractive Manufacturing
STL: Standard Tessellation Language
RCTs: Randomized Controlled Trials
SLM: Selective Laser Melting
PMMA: Polymethyl Methacrylate
Co-Cr: Cobalt-Chromium
OHIP-EDENT: Oral Health Impact Profile in Edentulous Adults
OHRQoL: Oral Health-Related Quality of Life
MPF: Maximum Biting Force
EDM: Electrical Discharge Machining
